# Carework and caring: A path to gender equitable practices among men in South Africa?

**DOI:** 10.1186/1475-9276-10-17

**Published:** 2011-05-09

**Authors:** Robert Morrell, Rachel Jewkes

**Affiliations:** 1 School of Education, University of Cape Town, Rondebosch, 7700, South Africa; 2 Gender & Health Research Unit, Medical Research Council, Private Bag X385, Pretoria 0001, South Africa

**Keywords:** Care, Masculinity, HIV prevention, gender equality, South Africa

## Abstract

**Background:**

The purpose of this study was to examine the relationship between men who engage in carework and commitment to gender equity. The context of the study was that gender inequitable masculinities create vulnerability for men and women to HIV and other health concerns. Interventions are being developed to work with masculinity and to 'change men'. Researchers now face a challenge of identifying change in men, especially in domains of their lives beyond relations with women. Engagement in carework is one suggested indicator of more gender equitable practice.

**Methods:**

A qualitative approach was used. 20 men in three South African locations (Durban, Pretoria/Johannesburg, Mthatha) who were identified as engaging in carework were interviewed. The men came from different backgrounds and varied in terms of age, race and socio-economic status. A semi-structured approach was used in the interviews.

**Results:**

Men were engaged in different forms of carework and their motivations to be involved differed. Some men did carework out of necessity. Poverty, associated with illness in the family and a lack of resources propelled some men into carework. Other men saw carework as part of a commitment to making a better world. 'Care' interpreted as a functional activity was not enough to either create or signify support for gender equity. Only when care had an emotional resonance did it relate to gender equity commitment.

**Conclusions:**

Engagement in carework precipitated a process of identity and value transformation in some men suggesting that support for carework still deserves to be a goal of interventions to 'change men'. Changing the gender of carework contributes to a more equitable gender division of labour and challenges gender stereotypes. Interventions that promote caring also advance gender equity.

## Introduction

Gender inequity is one of the major barriers to global development and the attainment of health for women [1,2]. In South Africa research points to a hegemonic masculinity, particular described among African youth but not restricted to them, that is predicated on prodigious demonstrations of success in acquisition and control women sexual partners, and hence is sexually risky and often very violent [3]. Research demonstrates that such a masculinity, and more broadly inequitable gender relations, underlie the problems of violence against women, risky (hetero) sexual practices and HIV [4-9]. These conclusions are increasingly translated into policy requiring that measures to promote more gender equitable models of masculinity (to 'change men') are included in development and HIV prevention approaches [9-12]. This focus on men presents a series of intellectual challenges, especially for the evaluation of interventions, as it begs the question: what is a gender equitable man? Is it a man who espouses gender equitable attitudes, or one who demonstrates gender equitable practices? If the latter, which practices contribute to gender equity or reflect commitment to gender equity?

Whilst violent, controlling and sexually inconsiderate practices towards women are agreed as indicators of gender inequity, there is a temptation to fall back on the absence of these as indicators of gender equity [e.g. [13]]. These practices have substantial health and social consequences for women, yet the position and use of violence in the establishment and maintenance of male hegemony is an on-going area of debate [14-17]. Indeed the notion of 'hegemony' itself implies ceded power, rather than power achieved over another through force, the use of violence is not inherent in hegemonic masculinity [14,15]. Importantly 'benevolent patriarchies' that are not violent are well recognized, and often gain power and legitimacy through their ability to 'other' the excesses of more violent masculinities. There is a strong argument that gender equitable men should be identifiable through their engagement in a range of practices that extend beyond the absence of the negative in relations with women and the use of violence. In this regard, a strong candidate practice is engagement in caring, previously seen as the terrain of women. Research on men's involvement in carework is growing [e.g. [18,19]]. Yet women dominate the care professions (teaching, nursing) and via motherhood, are generally held 'by nature' to be more caring than men [20]. Thus men's engagement with caring may be another indicator of being more gender equitable. If it is to be used in this way in evaluation of programmes to 'change men', it is important that we understand more about men's engagement in caring, its origins and the relationship between men's caring practices and ideas and other gendered practices.

Caring for others takes many forms. In addition to the caring professions, where men often top the professional hierarchies [20], caring for one's own children is a strongly gender biased area of domestic labour. One of the major areas of feminist engagement over many decades has been to address this unequal distribution of work [21]. Initiatives have been taken up in many countries to address parental inequities, notably the introduction and expansion of paternity leave in the Scandinavian countries [22].

In constructions of masculinity, care is most usually interpreted through expectations of protecting and providing for families, which is seen as a marker of good fatherhood practice [23,24]. Providing is a central feature of working class masculinity, though it goes together with patriarchal views more generally. In Africa, despite widespread poverty and unemployment, many men aspire to be good fathers, but fall short. Yet if the definition of caring is extended beyond provision and protection to include hands-on ministering to the sick, aged, young and infirm and an emotional engagement with those to whom care is provided, its place in the constellation of values that produce masculine identity changes. Caring practices and values proclaim a commitment to alternative interpretations of masculinity and hold some promise for gender equality and responsible, health-conscious, risk-averse behaviour.

The term 'alternative masculinities' refers to those constructed in opposition to hegemonic masculinity. The concept has been developed in a theoretical context in which Connell's framework [25], which includes hegemonic, complicit and subordinate masculinities, allows for the existence of men's identities and practices to express opposition to and distance from a hegemony which is predicated on the oppression of women and other men. We use it in this sense though note that masculinities often contain contradictory elements [26,27] which may well be supported by women who are complicit with patriarchy. Hegemonic values discourage men from expressing alternative masculinity [28] but equally can change to accommodate more equitable gender values while stopping short of challenging the fundamental structures of gender inequality [29]. Alternative masculinities can be promoted by gender interventions [30].

In order to explore how care is located in constructions of masculinity we present findings from qualitative research with South African men engaged in caring. South Africa is often associated with violent men and may seem a curious setting for research to document, theorize and politicize caring as an activity in which men engage. Its history of colonialism and apartheid and rates of homicide and rape are notorious, yet, in the context of research on fatherhood and on how men cope with their own vulnerability there is now a growing literature which documents and analyses men's caring [21,31,32]. This literature also argues that caring is a way of contributing to the realization of gender equality because it shifts 'women's' work onto men and unlocks in men, the desire to love and care for their children and dependents, their family and even for themselves.

In this paper we explore three questions: how were men engaged in caring? What are its origins? And how does it play out in their daily lives and identities? As we describe below, the study has a limited sample and makes no claim for generalisability. On the other hand, the cases we discuss provide detailed insights into how men engage in carework and relate this to their understandings of themselves as men. Both their practices and their gendered understandings have implications for gender equality. The data was initially collected as part of a multi-country project on 'Men who Care', which was part of the Men and Gender Equity Policy Project. Through this project our methodology has also been implemented in India, Brazil, Mexico and Chile.

## Methods

We interviewed twenty men, seven in KwaZulu-Natal Province (Durban), seven in and around Mthatha (a small town in the Eastern Cape) and six in Gauteng Province (Johannesburg and Pretoria) in 2008. The men ranged in age from the early 20s to the mid 80s. They included African, white and Indian men, Christian, Muslim and Hindu. In social class terms, they ranged from middle class, university trained professionals to the un- or marginally employed. The sample was purposive and we operationalised a loose definition of 'men who care'. We tried to select men who demonstrated care in three different, though possibly overlapping, ways. Our first category is men who were in paid carework that was ordinarily associated with women, for example, nursing or social work. Our second category was for men engaged in the bulk of parenting or childcare. In reality, this definition stretched to include men who took the lead responsibility for various forms of house and child care. The third category belonged to men working for NGOs or engaged in community work, either in a voluntary or paid capacity. We chose NGOs which were specifically involved in gender sensitive work. Our sample therefore included men working in various forms of HIV and AIDS support work, work to build gender equity, gay health, and child welfare. Numerically this meant that we had seven 'fathers', six NGO workers and seven men in professional carework (See Table 1). In terms of sexual orientation and other diversity, two informants were gay, one was disabled and two were living openly with HIV. We used snowballing and other forms of networking to identify interviewees. The slight over-representation of NGO workers reflects the greater visibility of men involved in NGO work than those who undertake a major or the sole responsibility for child and domestic care.

**Table 1:** Informants

Pseudonym	Type of Care	Social circumstances
1. Dumisani	NGO Activist, working with youth	40 years old, Zulu, straight

2. Pramesh	Primary school teacher and counsellor for NGO (Gay Health Club)	45 years, Indian, Hindu, gay (only partially open about this)

3. Jaz	Trainee Nurse	32 years, Indian, Muslim, gay, married to a man

4. Charles	Psychotherapist, NGO volunteer worker, university lecturer	Mid 50s, White, straight, married to a woman

5. Linda	Provider for 10 siblings and nieces & nephews, aged 1-17 years. Previously he supported 17 of them	23 years, Zulu, straight, marginally employed

6. Cedric	Retired doctor, volunteer worker for local NGO on gender and AIDS prevention	85 years, White, widower

7. Mzokhona	Single father to three infants	36 years, Zulu, straight, unemployed

8. Kabelo	Forensic Nurse, single father of 2 children	42 years, Sotho, straight, divorced

9. Xhanti	Health promotion and community development	30 years, Xhosa, straight

10. Jim	Works in Gender NGO, youth and childcare worker	37 years, White, married, straight

11. Steve	Foster father and primary carer for his children whilst his wife developed her career	Age about 45 years, White, straight, married

12. Neo	Worked for a gender NGO	About 50 years, African, straight, married. Disabled

13. Simon	Social worker for abused children	About 33, African, straight

14. Thulani	Very engaged in caring and contributing at home	Mid 20s, Xhosa, straight

15. Mzwandile	Active in support groups for people with HIV/AIDS & community work on violence against women	About 30 years, Xhosa, straight, HIV+

16. Mcebisi	Primary carer of his 6 year old daughter whilst his wife lives away studying nursing	Mid 40s, Xhosa, married

17. Pat	Volunteer worker, HIV care	Mid 30s, Xhosa, straight

18. Dennis	Cares for sick girlfriend with HIV	Early 20s, Xhosa, straight

19. Sipho	Involved in community activities, especially drama, on HIV and rape, and home based care	Early 40s, Xhosa, straight

20. Bonginkosi	Cares for mother and 7 nieces and nephews	Mid 40s, Xhosa, HIV+

In general, we utilized our existing networks to identify informants. For example, we contacted local Non Government Organisations with a profile in gender work and asked them to suggest potential informants. In some instances we used the media to lead us to informants. This was the case with Mzokhona, a father of triplets, who was celebrated on father's day by a Durban newspaper. Health practitioners were identified through our networks and by approaching practicing doctors and medical faculty and asking for leads. The identification of informants invariably required sleuth work to find their contact details and then to arrange a time and place to meet. Interviews were generally conducted at a place and time convenient to the informant. Each interview began with a discussion of the project and its goals, a process that was concluded with the signing of informed consent. An information sheet on the study was given to informants.

The nature of our sampling resulted in interviews with men who, it turned out, did not fit our preconceived ideas of 'men who care'. Some men had a 'reputation' for being unusual in the carework that they undertook, but turned out in fact to be more orthodox in terms of gender role (and attitude) than we expected. On the other hand, each one of the informants understood that he was, in terms of the local gender standard, unusual. Given that we used snowball sampling, this outcome reflects a diversity in how *others *view men who care and it seemed that 'caring men' were often perceived to be identifiable by demeanour, a softness of personality, rather that subscribing to a particular set of ideas or necessarily engaging in a particular set of behaviours. Even at this stage it was clear that men who cared were not monolithic exemplars of gender equality and that there was frequently a mismatch between roles as caring men and their more general behavior, including towards women.

The men were interviewed by the two authors and a Xhosa-speaking researcher. The interviews followed a modified life history approach, with men asked to talk about their lives from childhood, important influences in their lives and how they saw themselves as men, and how they became involved in the care activities that resulted in their selection in our sample. They were asked about their home life and relationships currently, how they relate to their family, and views on some of the gender equity laws of the country. Interviews were taped, transcribed and where necessary, translated. Most men had one interview lasting about 1.5-2 hours and three of the men had an additional follow-up two interview necessitated by time limitations that prevented completion of the first interview. Ethics approval was given by the Medical Research Council and names used are pseudonyms.

Data were analysed by the two authors, who both coded the interviews, initially into broad categories stemming from the scope of inquiry for the interviews. The initial codes were: caring relationships that men have, early (or first) manifestations of caring, relationships with fathers, mothers and significant adults in relation to caring, link between caring and own children, link between caring and gender equality, 'ideological' origins of/links with/to caring, caring and traditional attributes of masculinity, caring and paid work and sexual division of labour, the interviews were broadly coded and thereafter they were sub-coded into categories that emerged from the interviews. Using analytic induction mini-theories were generated and tested [33]. An example of this was a theory that men who cared had a significant, supportive adult family relationship in childhood. This was later modified with the observation that these were not necessarily family relationships and not described by all of the men, but by many, leading support to a conclusion that there were multiple paths to caring.

## Results

The men interviewed were engaged in a wide range of activities, summarised in Table 1. Although each was approached with an idea that he fitted one of the recruitment categories, it often became clear that caring took multiple forms in the men's lives. For example Kabelo was a nurse involved in gender work, a single father, and had been a carer at home from childhood. Jim worked professionally with NGOs on woman and child abuse, but had also fostered a baby alone and was very involved in voluntary work. Pramesh was a school teacher and voluntary counsellor. Several of the men were recruited because of NGO activity, for example Neo and Dumisani worked on men and gender equity. Cedric, aged 85, was much older than other informants and as a post-retirement activity became involved through a hospice, working with men on gender and HIV prevention. With the exception of Jim and Cedric, at the time of interview the NGO workers were mostly paid a small stipend (as little as R500 (US$60) a month) rather than a salary, but all of them had been unpaid for the greater part of their time involved in community activities.

The last group of men were recruited because they had major responsibilities for child care or other forms of domestic caring. Mzokhona, aged 36, was unemployed and the sole carer of his 2 year old triplets, after their mother left them on his doorstep in a cardboard box when they were 10 months old and disappeared. Linda was aged 23 and had a very low paid job. Alone, he looked after five siblings and five nieces and nephews aged 1-17 years. Bonginkosi began caring for his diabetic mother and looking after his seven nieces and nephews after he became ill with HIV. Some of these men clearly were shouldering responsibility of remarkable scale, others less so, but what they all had in common was that they had adopted a caring role that many other men (and sometimes women) from their social background would not have taken on. This raises a critical question, which is: why did they do it?

### Origins of caring

There was no single pathway into caring. Whilst men's narratives often gave particular emphasis to one influence, reflection on their lives rather suggested that the routes were marked by constellations of influences and interactions, which impacted to different degrees, sometimes cumulatively, on the often non-linear pathway to the present circumstances of care. Caring was deeply in-grained in the make up of some men, as they had been involved in caring activities from a very early age and some of them adopted alternative masculinities from childhood. For example Kabelo was looking after his younger sister and taking her to crèche from the time he went to school. Xhanti was sole care-giver for his younger brother from the age of 12 whilst his mother was away during the week working, and Simon (middle ranked among the children in age) cooked and ironed for his granny and 15 cousins and siblings from early in his childhood.

For other men, caring started later, and often they mentioned the influence of a particular family member, teacher or religious figure in their lives, or the influence of politics. Pramesh, who articulated a religiously-inspired life purpose as "to promote tolerance, and good values", was inspired by a Hindu guru, as well as his caring grandmother and mother, whilst Cedric was influenced by his grandfather, whose liberal teachings and charitable work laid lifelong foundations for his caring attitude towards others.

Having no father, Bonginkosi was influenced by a man who lived in the community who showed him great care, encouraged him and taught him respect. He grew up in a rural area, herding and hunting, he explains:

"if I had not met him and met up with friends, maybe I would have been involved in taking people's things [stealing] ...In my growing up, I have never stolen a goat. Guys who were my age were stealing goats, sheep and chickens. I stopped at stealing peaches and even there it was just a phase and I stopped... I grew up loving beautiful things, doing things with my hands, working with my hands. I did not like being dependent."

Some spoke of the impact of a life-changing event, such as a death of a relative, or, like Mzwandile and Bonginkosi, discovery that they were HIV positive. Jaz experienced a series of family tragedies when, first, one of his older cousins was shot in a robbery and died after Jaz drove him to hospital, and then his brother committed suicide. He explains:

"I am not a violent person at all, I am totally a passive person in all aspects, and I think from seeing all this violence around me as well, and stuff happening to my family as well, that I felt that nursing was the way to help this kind of situation."

The men from the NGO sector had mostly become involved in HIV and gender carework when unemployed, often because the opportunity presented itself for them to gain self-respect by being busy and useful. It wasn't always clear that it was a deliberate choice.

Xhanti was somewhat different from the other NGO men as he had a university degree and chose to go into HIV work, but he presented a narrative in which he described his gender transformation as coming thereafter. The trigger to his 'becoming caring' was exposure to the gender transformative HIV prevention intervention, Stepping Stones [34,35], which he attended (as did our interviewer Yandisa) as a series of workshops over about 50 hours. He explains: *"I see myself as different from other men...I am more accommodating and compassionate"*. Although his life had clearly involved carework before he attended Stepping Stones, the programme assisted him to re-frame his identity as a 'man who cares'. As he explained:

*"I was like other men before, until that time... I started to see things differently... I think it was at the workshops...and I think after that I changed"*.

Some of the men seemed to have been thrown into caring roles in the face of life circumstances, they didn't always frame it as a choice, although to the extent that they often picked up where others had abandoned, it clearly was a choice of sorts. Mzokhona was one example. He was unexpectedly left to care for his 10 month old triplets. He lived alone in a rough shack with no family or state support. His situation as a single father was highly unusual as most men would either pass the childcare burden on to female family members or simply walk away from their paternal responsibilities. Likewise Linda was left in charge of the children when other adults either died, or starting with his father when he was 10, just left home: *"My grandmother passed away (2001). I stayed with my mother. My mother decided to leave. My mother got married and went away (in 2004) with the husband. My sister drank too much. I was staying with her in Lamontville. And she could not cook for those children. And she went away."*

Dumisani, who saw providing protection as central to his caring, was an underground ANC activist from the age 14 years when he had entered politics to protect his activist younger brother. He equated caring with being a warrior, protecting his community: *"There was no way one could not be part of the violence, whether it was either perpetrating it, with the group you were part of, or sometimes one would get involved defending oneselves. There was no way one could shy away from it."*

Many of them had strong caring female or male role models in their lives, but these were often not their parents. Whilst there is an abundant literature linking childhood adversity with men's later violent and antisocial behaviour [[36], Mathews S, Jewkes R, Abrahams N: **'I had a hard life': Exploring childhood adversity in the shaping of masculinities among men who killed an intimate partner in South Africa**, submitted], these men's childhoods were diverse, and some that were notably harsh. An example here was Mzwandile who, with his brothers, was abandoned by both his parents after their marriage broke down. They were raised in the homes of aunts and uncles who beat them, starved them and used them as unpaid child labour in their business (this is not uncommon in such alternative care arrangements in South Africa [Nduna M, Jewkes R: **Disempowerment and distress in the lives of young people in Eastern Cape, South Africa**, submitted]. The cruelty was mitigated by the strong positive role model and care ultimately provided by his elder brother, and later by the influence of an older woman in whose house he lodged when at school. His brother, who had managed to find employment in the army, was eventually able to provide for his two siblings, give them a home and send them to school. In stark contrast to a classical military masculinity, Mzwandile described his brother as a person who was 'very calm' and would cry when he became angry and would always promote 'surrender' in an argument rather than fighting.

Many of the men grew up without contact with their fathers. Some had died, some had not been disclosed to them, and others knew who they were, but had no contact. Some fathers had rejected their mothers, and by implication them. On the other hand, some of the men came from conventional nuclear family homes with two parents who loved each other. Jim described his father as "*a very quiet, caring presence*" who gave him a great deal of support and inspiration.

So whilst it could be argued that the men had been shaped in their ideas of caring through being able to form strong attachments with influential adults, it was also apparent that many of the men we interviewed had very actively sought out and developed relationships with adults where their siblings hadn't. This was apparent in the narratives of engagement with mothers or grannies in domestic work. In Mzwandile's family, the middle brother became a hardened gangster and never connected with the caring influence of the oldest one. Possibly it came too late for him. Thus it appeared that in childhood, as well as later life, the men who cared both cared for others, and seemed to have actively sought relationships in which they received care from others, even in some cases in circumstances of extreme adversity. The impact of social support on psychological resilience in the face of stress and adversity is well-described in the literature [e.g. [37]]. The narratives of forming supportive relationships from men in our study, particularly those from families where they developed strong relationships and other siblings didn't, leave open the question as to whether these men were merely 'fortunate' in having access to these, or whether people who offer such support are more likely to do so for boys or men who are seen by nature to be more 'caring'.

### How do men themselves narrate care to broader issues of masculinity?

For some men caring was a duty to protect and fitted comfortably with conventional masculine ideals. For others caring was expressed primarily as emotional engagement and went far beyond being a provider and protector. These men explained their carework in a way that drew on and contributed to gender equality discourse. None of the men saw their caring as 'unmasculine' and they all conveyed a confidence in their gender identity and practices which allowed them to accommodate apparent contradictions.

Some of the men very explicitly rejected the ideals that they recognized as reflecting hegemonic masculinity (occasionally using this language) and saw their caring as part of an alternative, gender equitable masculinity. Among them were all of the men who were involved professionally with carework or who worked with a gender NGO. For example, in addition to his professional work as an academic, Charles has always believed in sharing house work and undertook this work from the start of his marriage. He mused about his own masculinity: *"I have in some ways grown up on the edges of hegemonic masculinity, so I have been less inclined to identify with hegemonic patterns. So it has been easier I think, for me, to slip into a role of gender equity, than it might have been if I had a very strong hegemonic identity."*

Similarly Kabelo had practiced an alternative masculinity that embraced gender equity and non-violence towards women well before becoming involved in gender issues at work. He had cared for his sister as a child, challenged his father's use of violence and looked after his eldest child (son) from the age of one because the child's mother went away to train as a teacher. He explained: *"People sometimes laugh at me because I would carry him on my back from my parents' place." *But confident in his alternative masculinity, this didn't worry him. Very unusually for South Africa, he gained custody of his two children after his divorce.

For Xhanti, his alternative masculine practices emphasized blending care and respect for women: *"I listen to people usually, and I don't interrupt when they have arguments. You find other men, they will boo when women argue, so maybe I apply the skill differently, and I take their views. I think I am different." *He emphasized these over, for example, shared domestic duties. He was happy to do his own cleaning, cooking and washing, but when his girlfriend (the mother of his child) was around, she would take over responsibility. "*I agree it is something to be changed*", he laughed, acknowledging the contradiction with ideals of gender equity.

Simon indicated how his grandmother had persuaded him to become her 'special helper' and protector, and thus introduced baking and ironing in a way that would not make his feel unmanly. He grew up being very conscious that he was doing things his male peers didn't do, but did not seem to mind:

"she would tell me here at home we are protected by you, if someone will attack us its got to be you who protects us, but on the other side she said I had to do household chores cook, bake and do all that thing, ironing...I always liked those things that males don't do. I used to enjoy cooking and baking on a Sunday... I learnt a lot from my grandmother."

Secure in the adoration of his grandmother, he presented his caring as part as a secure, alternative 'soft' masculinity, explaining *"I am a male, although I am a little bit soft."*

For most of the men interviewed from Mthatha, caring was variously interpreted as providing protection (including of women from violence), being independent, and responding to circumstances necessitating care at home. In some of these interpretations, it sat fairly comfortably within local understandings of patriarchal masculinity, although most of the men perceived themselves to be different from and better than their peers and rejecting of some of the common male practices characteristic of hegemonic masculinity. For example Thulani didn't support all the gender equality laws, though he was generally a caring person who felt that he was different from other men because he'd *"never been interested in being involved with more than one female for the sake of having many girlfriends"*. Mcebisi, for example, said *"I am a person who sits with my family most of the time. I do not have time for friends. It's my family or I go home or I go to church"*. He was particularly proud of having a very good relationship with his daughter.

Dennis professed to care for others, but he hardly mentioned his own children at all. Almost as an afterthought at the end of a long interview he added: *"I have an experience about that, I have a kid, and in fact I have them, they are two. It's a boy and a girl. It's first the girl then comes the boy"*. His children were not priorities in his life and he seemed to be repeating his father's absence from his own childhood. Yet Dennis distinguished himself from other men by a curious code of public fidelity, through which he told us he was a decent man: *"I am able to stick to one person. That is why when I am going out with a person; I am this person who gets her pregnant because I am able to stick to one person. It's not that there are no others, but those that are around, she won't be able to see them. She gets her place as much as she wants." *Dennis argued that he allowed a woman to be secure with him because he acknowledged their presence in public and thus saved them from being mere clandestine girlfriends (*khwapheni*).

Whilst viewing themselves in some respects alternative (and being viewed as such by others, hence their recruitment to our sample), and being more caring, less violent, more faithful, than other men, many of the Mthatha men drew on masculinist discourses to explain themselves. Bonginkosi, for example, said *"Most of the times I do not want to bow down a lot to a person. I like doing things for myself and it is nice if I do something by myself." *A man must be independent and strong. In Mthatha, independence may overlap with engagement in a range of domestic chores which in a marital context would be viewed traditionally as 'women's work', but actually are essential for men to engage in if they are to be independent. Their alternative practices were expressed generally as making them 'better men', often more dutiful to the family, but were not framed in terms of gender equity.

Some Mthatha men, like Mcebisi, expressed views that were classically patriarchal. He believed that *"fathers are taught to take responsibilities in their houses"*. Bonginkosi described women as abusive, loud, extractive, devious and overbearing, and believed men were better carers than women. After an illness and being diagnosed with HIV, he was unemployed and looked after seven nieces and nephews and his mother who had diabetes. He saw himself as the rock of the family who kept it together, protecting his mother from a burden placed on her by her irresponsible daughters. He explained:

"[I]t is better that I stay here at home and whatever I am trying, I should try it closer to my mom. I like to see her happy even if it's not much happiness, but sometimes I can see that she is feeling free, she is alright... she must not think that she is left with the kids because of her daughters."

### What is the relationship of 'care' to 'gender equality'?

Caring could be interpreted as duty, or an emotional and political commitment. Men who undertake carework out of duty were perhaps a minority in South Africa where most men eschew caring work altogether and take family responsibility lightly. The men in this study proudly presented their caring activities as reflecting a masculinity that was morally superior to such men, as well as to women. Regarding themselves as responsible and good role models, in the discharge of the 'duty' of care, they affirmed a version of masculinity that was in some respects at variance with local norms, although not traditional ideals. Care as a 'duty' was generally of the 'provider' and 'protector' variety and involved quite traditional masculine understandings of gender roles, albeit reflecting a benevolent patriarchy. Thus many men retained patriarchal beliefs which included that men were naturally superior to women and that men should make decisions. Allied to these views were a battery of beliefs that are globally associated with aggressive patriarchy, including homophobia, punitive punishment systems and rigid authority structures (often supported with reference to biblical texts). All of which question the extent of the 'benevolence' of this patriarchy. This category of 'men who care' had not had any formal training or education in the value of gender equality, and had not been exposed to the influence of people who espoused feminist ideals. Necessity had propelled them into caring, their engagement with it had engaged them to view themselves as 'good men', but there was little stimulus to embrace the principles of gender equality.

Men who viewed carework as a choice, part of a political commitment to fairness, and an affirmation of an alternative masculinity, were much more likely to embrace gender equality. Here carework was not limited to providing financial or other resources nor did it involve them in occupying a decision-maker or authority position vacated by somebody else. Carework was rather framed as an integrated commitment to a set of values, which were often explicitly opposed to the use of violence, patriarchy and authoritarianism. A central distinction here was emotional engagement with the notion of caring and embracing of an identity as being 'men who care'.

## Discussion

Is male carework a constituent element of a path to gender equity? Our answer in this paper is both yes and no. On the one hand, when men engage in carework as practice they challenge gender norms by showing that men can play a role in an area of work generally regarded as the responsibility of women. In this sense carework contributes to gender equality by challenging the unequal gendered division of carework and, consciously or not, they may serve as role models for other men. Some, but not all, men also brought to their carework an explicit commitment to gender equality which spread far beyond the realm of the work itself. This was often explicitly expressed as opposition to gender oppression or at variance with gender unequal practices and attitudes. These men very clearly sought to align their activities and their ideals. Yet our study has also shown that carework can also be framed by men in a way that is congruent with patriarchy and patriarchal ideals of gender roles, albeit without its violent expression. Some of the men either openly rejected gender equality as a goal or a vision of a future society, or endorsed various practices and values which ultimately entrench or express the power of men.

The concept of hegemonic masculinity attempts to capture the mix of practice, ideal and attitude among men which reflects the situation of gender inequality that is frequently identified with and by the shorthand term 'patriarchy'. In this article we have argued that a form (or forms) of masculinity that challenges or undermines hegemonic masculinity can be identified among the sample of men who care discussed in this article. The concept and the way it is utilized in research is the subject of vigorous and ongoing debate [15]. We have used it to refer analytically to practices and attitudes identified in South Africa as reflecting gender inequality, but also of perpetuating it. Gender inequality occurs both in the public and private spheres. In South Africa prime examples of inequality are high levels of gender based violence, inequality in employment and payment trends, and in attitudes relating to gender hierarchy and gender roles [38,39]. Hegemonic masculinity as concept can be used to show how men collectively, across race, class, age, religious and other social divisions, broadly endorse the power of men. In heterogenous societies like South Africa with long histories of racial segregation, it is possible to argue the existence of multiple hegemonies, whilst acknowledging commonalities, including the idea either of male power that is central to theories of patriarchy, and that diverse groups of men may have a similar interest in upholding power and in opposing gender equality [40].

It is in the context of men supporting patriarchy that we have in this paper asked questions about carework practice becoming the basis for alternative masculinities that embrace a vision of gender equality. In this vision, not only are inequalities between men and women addressed but the ways in which men go about their lives are also the objects of change. The development of alternative masculinities is critical for the achievement of gender equality.

The men interviewed were largely regarded by themselves, or by others, as expressing alternative masculinities, although being 'alternative' appeared to lie on a spectrum, spanning a broader range of practices and ideas for some men than others. Many had multiple features that would single them out as 'different' from other men, whereas for others the predominant 'alternative' feature was their caring responsibilities and practices. Although fulfilling a provider/protector role was a much narrower interpretation of caring, by embracing caring in a context of poverty, violence and unemployment, they were considered by others to be expressing an alternative masculinity. Many South African men do not accept they have an obligation to provide; for example, research shows that only about half of children are financially supported by and in regular contact with their fathers [41,42]. Only some of the men themselves equated their caring and difference with being more gender equitable.

When considered relationally, the alternative dimensions of the masculinity of men interviewed did not simply align with the values of gender equality. For those who were professionally and politically involved in carework, there was a much more visible alignment with gender equity, than for others. A number of influences and interfaces in their lives had contributed to this. Important here was having a strong male or female role model who through their example, advice or actions gave space for the expression of alternative masculinities. We noted that exposure to ideas of human rights and notions of 'fairness' were as important as explicit engagement with feminism (understood by them as women having the same domestic status and the same rights as men). For some men, illness or trauma enabled them to reflect on their values and the lives they wanted to lead, and to express alternative masculinities. One had been given the opportunity to reflect on his gender position by exposure to a gender transformative intervention.

Men who emphasised an emotional dimension to their caring showed generally a better alignment with gender equity. Yet there was evidence that even in cases when caring had been commenced as 'duty', a process of reconfiguration of identity and values had commenced and led to broader (if incomplete) change in some men. While many behaviour change theories, such as the Theory of Reasoned Action [43] are based on an assumption that attitude change precedes change in practice, others including Social Cognitive Theory acknowledge that changes in practice can result in changes in attitude [44]. Our findings provide some evidence to support the latter, yet we acknowledge that there is a danger that if definitions of 'caring' and being 'good men' include men who occupy more benevolently patriarchal positions, there is a risk of reifying this masculinity, and thus potentially further entrenching male privilege. Given that our study has shown that there was no linear relationship between providing care and gender equity, we suggest that efforts to change men through a focus on caring should secure a connection with gender equity through promoting human rights, empathy and emotional involvement rather than just caring as duty.

This study was small and exploratory and as a qualitative study it is impossible to generalize from the findings to all men who care. We have sought to provide insights into the relationship between caring and gender equity through interviews with a purposively selected sample of men who were largely known or connected to networks of the three interviewers. This will have introduced a range of biases, and will have influenced the representations men gave of their lives in the narratives, particularly the possibility of these being socially acceptable accounts. Yet given the nature of data and the conclusions derived, we believe that any such biases will not detract from the overall conclusions of this work and our assessment of their implications for the work of others.

## Conclusion

Men find their way into carework for many different reasons. While this carework is, in and of itself, important and should be encouraged, it should not be seen as synonymous with or as necessarily associated with commitment to the goals of gender equality. Perhaps this does not entirely matter. Men should be encouraged to engage in carework in order to provide support for children, the aged, partners and those in ill-health, to address inequalities in the gendered division of labour and to challenge stereotypes that associate carework with women. Whatever the motivation, it is fair that men share the burden of care and support those who need care. There is also a likelihood that engagement in carework practices will result in a process of identity and value transformation, and whether or not it is the goal of individual men, it will contribute to wider societal processes of achieving gender equality. This includes developing alternative masculine practices which are themselves constitutive of broader processes of gender transformation. Thus men's engagement in carework deserves to be an explicit goal of interventions aimed at 'changing men' and, although far from a perfect indicator of gender equitable ideals and practice, it is valuable in its own right as an outcome for such interventions.

## Competing interests

The authors declare that they have no competing interests.

## Authors' contributions

This paper is a jointly researched and written paper. RM was the South African leader of the 'The Men and Gender Equity Policy Project'. Together with RJ he developed the methodology and coordinated the primary data collection. RJ and RM collected primary data respectively in Gauteng and KwaZulu-Natal. The paper was a collaboration from start to finish. The expertise of RJ and RM in health interventions and masculinities respectively fed into the paper.

Both authors read and approved the final manuscript.

## Funding

This work was supported by the MacArthur Foundation.
